# Epidemiological Insights into Autoimmune Bullous Diseases in China: A Comprehensive Analysis

**DOI:** 10.1007/s44197-024-00277-7

**Published:** 2024-07-22

**Authors:** Zihua Chen, Lanting Wang, Li Ma, Fanping Yang, Shengan Chen, Jin Yang, Haiqing Gao, Chang Tang, Ying Zhao, Zhen Zhang, Lin Tang, Haiyu Xue, Jian Ying, Yu Xu, Wenhong Zhang, Lingyun Shao, Hanqiu Liu, Xiaoqun Luo

**Affiliations:** 1 Department of Allergy and Immunology, Department of Dermatology, Research Center of Allergy and Diseases, Huashan Hospital Affiliated to Fudan University, Fudan University, 12 Middle Urumqi Road, Shanghai, 200040 P.R. China; 2 Department of Infectious Diseases, Huashan Hospital Affiliated to Fudan University, Shanghai, China; 3 Department of Radiology, Huashan Hospital Affiliated to Fudan University, Shanghai, China

**Keywords:** Autoimmune bullous disease, Pemphigus, Pemphigoid, Interstitial lung disease, Mortality, Triggers, Risk factors, Comorbidity

## Abstract

**Objective:**

This study aims to conduct an extensive analysis of autoimmune bullous diseases, particularly pemphigus vulgaris and bullous pemphigoid, in Shanghai, China, from 2016 to 2023. It seeks to understand the demographic profiles, comorbidities, mortality rates, risk factors, and socioeconomic impacts associated with autoimmune bullous disease.

**Methods:**

A cross-sectional study design was employed, enrolling 1,072 patients. Diagnostic measures included clinical manifestations, histopathology, direct immunofluorescence, and serologic tests. The study also involved a detailed socioeconomic analysis and evaluation of occupational risks.

**Results:**

The findings highlight a significant occupational risk in industries requiring enhanced safety measures, with a notable prevalence of autoimmune bullous disease among workers in these sectors. A considerable portion of the patients were from low-income backgrounds with limited literacy, indicating the economic burden of autoimmune bullous disease. A key discovery of the study is the potential pathological link between autoimmune bullous disease and interstitial lung disease.

**Conclusion:**

This research, one of the first comprehensive studies on autoimmune bullous disease in China, underscores the need for targeted healthcare strategies and further investigation into autoimmune bullous disease, particularly its relationship with interstitial lung disease.

**Supplementary Information:**

The online version contains supplementary material available at 10.1007/s44197-024-00277-7.

## Introduction

Autoimmune bullous diseases encompass a spectrum of severe dermatological conditions characterized by the formation of blisters on the skin and mucosal surfaces. These disorders arise due to autoantibodies targeting specific components within the cutaneous desmosomes or hemidesmosomes, leading to a loss of cell-to-cell adhesion and subsequent blister formation. Pemphigus and pemphigoid are the predominant forms of autoimmune bullous disease, each with distinct clinical and histopathological features.

Pemphigus, identified as an intraepidermal blistering disease, is marked by the development of superficial flaccid bullae that easily rupture, causing painful erosions. A hallmark of pemphigus is the positive Nikolsky sign, where gentle pressure on unblistered skin leads to exfoliation of the outer layers [1]. Histologically, pemphigus is characterized by suprabasal acantholysis, frequently precipitated by autoantibodies against desmoglein 1 or 3, and direct immunofluorescence typically reveals the deposition of immunoproteins between epidermal cells [2].

Contrastingly, pemphigoid presents as a subepidermal blistering condition characterized by tense bullae that do not rupture as readily, and the Nikolsky sign is generally negative. Autoantibodies target bullous pemphigoid antigen 180 or 230 within the hemidesmosome, leading to separation at the dermal-epidermal junction. The histopathological hallmark of pemphigoid is subepidermal blisters with eosinophil infiltration, and direct immunofluorescence often shows linear deposition of immunoproteins along the basement membrane. Notable subtypes of pemphigoid include bullous pemphigoid, mucous membrane pemphigoid, anti-p200 pemphigoid, and pemphigoid gestationis, each with unique clinical implications and management challenges [3].

The global epidemiology of autoimmune bullous disease indicates variability across populations, with some regions reporting higher incidences than others. Studies have demonstrated a greater cumulative incidence of bullous pemphigoid in Europe compared to Asia [4], while pemphigus, albeit less prevalent, also shows regional variation. Notably, the incidence of pemphigoid has been increasing, particularly in Western countries, over the past two decades. However, comprehensive data on the incidence and prevalence of autoimmune bullous disease in Asia, especially China, are still scarce [5–7].

Mortality associated with autoimmune bullous disease varies, with first-year mortality rates and standardized mortality ratios differing significantly by country and disease subtype. Pemphigus vulgaris and pemphigus foliaceus have been linked to a higher risk of death compared to bullous pemphigoid [8–10]. Yet, the specific mortality profile of autoimmune bullous disease within the Chinese population has not been extensively investigated.

Autoimmune bullous disease imposes significant health and financial burdens, including impact on quality of life and productivity losses due to the disease. The annual medical expenses for pemphigus patients were notably higher than for those without the condition [11, 12]. Treatment involves systemic steroids, topical hormones, and occasionally adjuvant immunosuppressive therapies [13]. However, long-term use can result in severe side effects [14]. Currently, immunotherapy shows promise in treating autoimmune bullous disease. For instance, Rituximab targets CD20 on B cells, depleting desmoglein -specific IgG-positive B lymphocytes in pemphigus treatment [15]. Dupilumab, an IL-4Rα antagonist, is effective in pemphigoid by inhibiting IL-4 and IL-13 signaling [16, 17]. These novel therapies offer opportunities for improved treatment outcomes, but further research is needed to optimize efficacy, safety, and assess long-term outcomes.

Considering the increasing morbidity, high mortality, and significant healthcare burden of autoimmune bullous disease, addressing treatment and early intervention strategies post-diagnosis is crucial. Epidemiological data are vital in shaping these strategies and guiding healthcare providers in their decision-making processes. This is particularly pertinent in China, where the aging population could influence the epidemiology of autoimmune bullous disease. Our study aims to provide a comprehensive analysis of autoimmune bullous disease clinical profiles within a Chinese context, offering insights to enhance the global understanding of these complex diseases and inform future therapeutic strategies.

## Materials and Methods

### Study Population

**Fig. 1:**
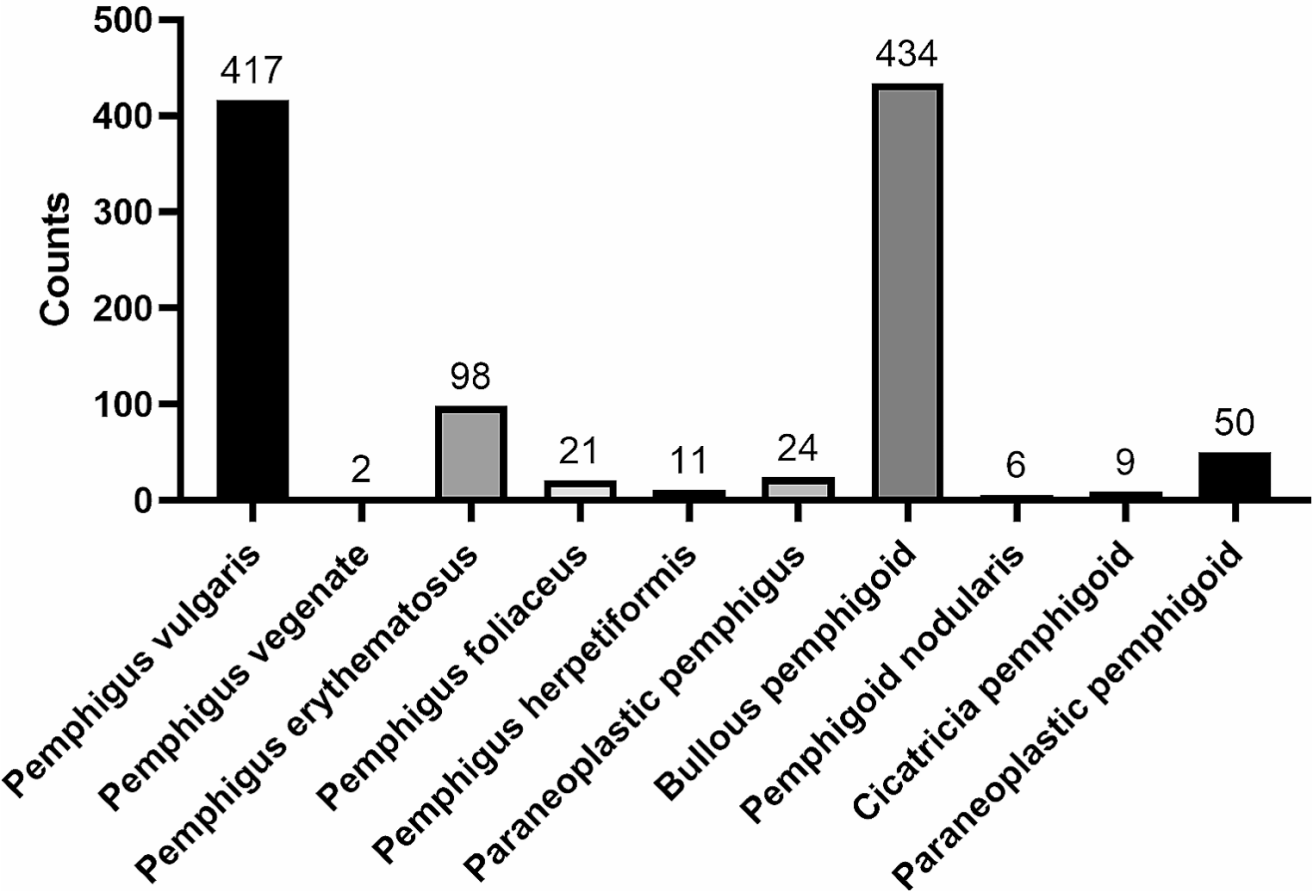
Distribution of autoimmune bullous disease types in China, 2016–2023. The bar chart illustrates the counts of patients diagnosed with various types of autoimmune bullous diseases. Pemphigus vulgaris (417 patients) and Bullous pemphigoid (434 patients) are the most prevalent conditions observed in this study. Other conditions, such as Pemphigus vegetans, Pemphigus foliaceus, Pemphigus herpetiformis, Paraneoplastic pemphigus, Erythema multiforme-like pemphigus, IgA pemphigoid, Cicatricial pemphigoid, and Paraneoplastic pemphigoid are represented with significantly lower counts, indicating a lesser prevalence in the study cohort. The numbers atop each bar indicate the total count of cases for each disease type

This cross-sectional study, conducted from September 2016 to March 2023 in Shanghai, China, focused on the diagnosis of autoimmune bullous disease based on clinical manifestations, skin histopathology, direct immunofluorescence assays, and serologic test results. Pemphigus vulgaris and bullous pemphigoid are identified as the two major types of autoimmune bullous disease. The diagnosis of pemphigus is established through: (1) histopathological evidence of suprabasal splitting and acantholysis; (2) direct immunofluorescence detection of immunoprotein deposition between epidermal cells; and (3) enzyme-linked immunosorbent assay identification of circulating autoantibodies against desmoglein 1 and/or 3. For bullous pemphigoid, the diagnostic criteria include: (1) histopathological presence of subepidermal blisters with eosinophil infiltration; (2) direct immunofluorescence detection of linear immunoprotein deposition along the basement membrane; and (3) enzyme-linked immunosorbent assay detection of circulating autoantibodies against bullous pemphigoid antigen 180 and/or 230 [18, 19]. The distribution of various types of autoimmune bullous disease is depicted in Fig. 1. All patients diagnosed with autoimmune bullous disease at our hospital, who provided informed consent, were included in the study.

Interstitial lung disease is characterized by inflammation or fibrosis within the interstitium, leading to dyspnea and, in many cases, respiratory failure and death [20] Lung imaging showed ground glass opacity or honeycomb appearance. To identify interstitial lung disease required multidisciplinary diagnosis, with cooperative evaluation by specialists from respiratory and radiology department [21]. Patients were considered ineligible if they had primary lung diseases. This included: (1) lung diseases resulting from various microbial infections, such as community-acquired pneumonia and tuberculosis; (2) chronic obstructive pulmonary disease; (3) silicon lung; and (4) long-term use of pulmonary toxic drugs.

Clinical data including demographic characteristics, socioeconomic profile, medical history, clinical symptoms, and laboratory parameters, were all collected at each outpatient visits and telephone follow-up to ensure accuracy and reliability. For socioeconomic variables, we cross-checked from patient self-reports and employment records to minimize discrepancies. To maintain data accuracy, standardized protocols were followed during data collection, ensuring consistency and completeness across all variables.

1072 patient with autoimmune bullous disease who met the diagnostic criteria were enrolled in this study. Each patient had been informed the purpose and content of the study before being enrolled. The study was approved by the institutional ethics committee of Huashan Hospital, affiliated with Fudan University (KY2016-398).

### Statistical Analysis

Data analysis was performed by SAS9.4 software. Qualitative variables were described as frequency and percentage. Quantitative variables were expressed in mean ± standard deviation (SD) or median and interquartile range (IQR). Chi-squared test was used to examine statistical differences between groups. Student’s t-test or Mann–Whitney U tests was applied to investigate the difference between quantitative variables. Cox analysis and Kaplan-Meier analysis were used to find the risk factors. P-value < 0.05 (two-tailed) was considered statistically significant.

## Results

### Demographic Profiles

**Fig. 2:**
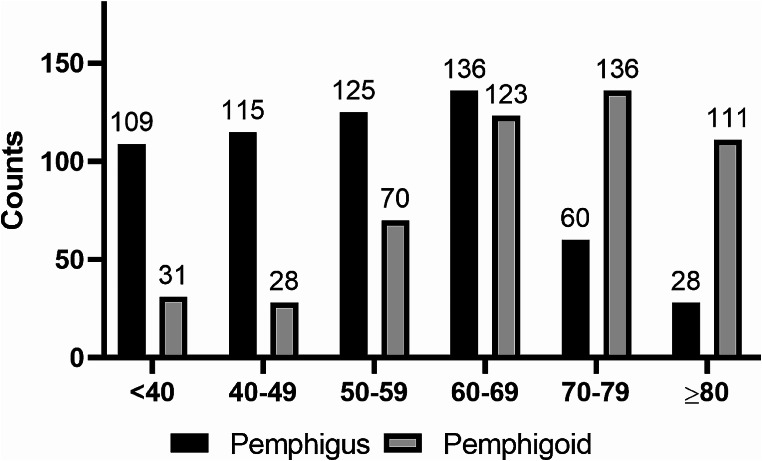
Age distribution of newly diagnosed autoimmune bullous diseases (AIBD) in China, 2016–2023. The histogram represents the number of patients with pemphigus (black bars) and pemphigoid (white bars) across different age groups. The age ranges are divided into six categories: under 40, 40–49, 50–59, 60–69, 70–79, and 80 years and older. The count of patients within each category is displayed above the corresponding bars. Notably, the age group 60–69 has the highest incidence for both pemphigus (123 patients) and pemphigoid (136 patients), followed by a marked decrease in the 80 + category

From 2016 to 2023, our study enrolled 1,072 patients diagnosed with autoimmune bullous disease, of which 573 were pemphigus cases and 499 pemphigoid. The cohort comprised 583 males (54.4%) and 489 females (45.6%), resulting in a male-to-female ratio of 1.19:1. Gender distribution showed no significant statistical difference. The average age at onset for pemphigus was 53.90 years (SD ± 15.62, range 10–94 years), and for pemphigoid, 67.31 years (SD ± 16.14, range 5-100 years), a difference that was statistically significant (*p* < 0.001). The incidence of pemphigus peaked between 40 and 60 years, while pemphigoid peaked between 60 and 80 years. The average follow-up duration was 4.85 years (SD ± 2.98). Detailed demographic data are presented in Tables 1 and 2; Fig. 2.

**Table 1:** Descriptive characteristics of patients with autoimmune bullous diseases (AIBD) in China 2016–2023

Variables	AIBD (n = 1072)	Pemphigus (n = 573)	Pemphigoid (n = 499)	*P*
Demographic				
Age at disease onset (years), mean (SD)	60.14 (17.21)	53.90 (15.62)	67.31 (16.14)	0.000
Male	583(54.40)	308(53.80)	275(55.10)	0.656
Female	489(45.60)	265(46.20)	224(44.90)	
Total mortality rate during the follow up	59(5.50)	22(3.84)	37(7.41)	0.051
Comorbidities				
Diabetes mellitus	166(15.49)	82(14.31)	84(16.83)	0.255
Cardiovascular disease	367(34.24)	160(27.92)	207(41.48)	< 0.001
Pulmonary disease	184(17.16)	90(15.71)	94(18.84)	0.175
Digestive system disease	113(10.54)	56(9.77)	57(11.42)	0.38

SD: Standardized Deviation

**Table 2:** The demographic feature of patients with autoimmune bullous diseases (AIBD) in China 2016–2023

Variables	AIBD (n = 1072)	Pemphigus (n = 573)	Pemphigoid (n = 499)	*P*
Age at disease onset (years), mean (SD)	60.14 (17.21)	53.90 (15.62)	67.31 (16.14)	0.000
Age (years), n (%)				0.000
<40	140 (13.10)	109 (19.00)	31 (6.20)	
40–49	143 (13.30)	115 (20.10)	28 (5.60)	
50–59	195 (18.20)	125 (21.80)	70 (14.00)	
60–69	259 (24.20)	136 (23.70)	123 (24.60)	
70–79	196 (18.30)	60 (10.50)	136 (27.30)	
≥80	139 (13.00)	28 (4.90)	111 (22.20)	
Gender, n (%)				0.656
Male	583(54.40)	308(53.80)	275(55.10)	
Female	489(45.60)	265(46.20)	224(44.90)	
Years of follow up, median (SD)	4.85 (2.98)	5.42 (3.09)	4.19 (2.71)	0.000
Total mortality rate during the follow up, n (%)	59 (5.50)	22 (3.84)	37 (7.41)	0.051
1 year mortality rate (%)	3.7	2.1	5.6	0.002
2 year mortality rate (%)	4.9	3.1	7.0	0.004
3 year mortality rate (%)	5.3	3.7	7.2	0.01

SD: Standardized Deviation

IQR: Interquartile Range

### Socioeconomic Status

Among autoimmune bullous disease patients, workers emerged as the most common occupational group, accounting for 33.16% of cases in both pemphigus and pemphigoid, closely followed by farmers at 31.46%. Within the worker subgroup, metal workers were predominant, comprising 14.55% of the autoimmune bullous disease population. The average annual income of autoimmune bullous disease patients was ¥36,000 (interquartile range ¥10,000–60,000), which is lower than the Shanghai average (¥58,988) but comparable to the Eastern China average (¥33,414). Notably, 28.64% of the patients belonged to low-income or non-income households. Additionally, 16.79% were illiterate, indicating an overall lower education level compared to the general population. These details are further discussed in Table 3.

**Table 3:** The socioeconomic status of patients with autoimmune bullous diseases (AIBD) in China, 2016–2023

Variables	AIBD (*n* = 1072)		Pemphigus (*n* = 573)	Pemphigoid (*n* = 499)	*P*
Occupation, n (%)					0.256
Peasant	262 (24.44)		147 (25.65)	115 (23.05)	
Worker	347 (32.37)		190 (33.16)	157 (31.46)	
Metal worker	156 (14.55)		90 (15.71)	66 (13.23)	
Chemical worker	61 (5.69)		31 (5.41)	30 (6.01)	
Transport worker	44 (4.10)		25 (4.36)	19 (3.81)	
textile worker	33 (3.08)		16 (2.79)	17 (3.41)	
Other worker	53 (4.94)		28 (4.89)	25 (5.01)	
Others	418 (38.99)		236 (41.20)	227 (45.59)	
Education, n (%)					0.276
Illiteracy	180 (16.79)		89 (15.53)	91 (18.24)	
Primary school	147 (13.71)		83 (14.49)	64 (12.83)	
Junior high school	362 (33.77)		205 (35.78)	157 (31.46)	
Senior high school	225 (20.99)		110 (19.20)	115 (23.05)	
College and above	158 (14.74)		86 (15.01)	72 (14.43)	
Personal year income (Yuan), median (IQR)	36,000 (10,000–60,000)		30,000 (1600–54,000)	36,000 (13,200–60,000)	0.014
Personal year income (Yuan), n (%)					0.001
<20,000	307(28.64)		174(30.37)	133(26.65)	
20,000–39,999	291(27.15)		172(30.02)	119(23.85)	
40,000–79,999	337(31.44)		153(26.70)	184(36.87)	
80,000–99,999	53(4.94)		22(3.84)	31(6.21)	
≥100,000	84(7.84)		52(9.08)	32(6.41)	

IQR: Interquartile Range

### Comorbidity Profiles among Autoimmune Bullous Disease Patients

Our study identified frequent associations of autoimmune bullous disease with cardiovascular diseases (34.24%), diabetes mellitus (15.49%), and pulmonary diseases (17.16%) (Table 4). Before the onset of autoimmune bullous disease, certain conditions were significantly correlated with pemphigoid, including cerebral infarction, dementia, Parkinson’s disease, encephalatrophy, asthma, psoriasis, ophthalmic disease, and type 2 diabetes (*p* < 0.05) (Table E1). Post-diagnosis, lung disease emerged as the most prevalent comorbidity in autoimmune bullous disease cases, accounting for 12.97% of the cohort (Table E2). Among the 79 cases with neoplasm, digestive tract tumors (23 cases, 2.15%) and lung cancer (19 cases, 1.77%) were most common. Lung and urinary system tumors were more frequently observed in pemphigoid patients (*p* < 0.01). Additionally, two cases of Castleman’s disease, developed during adolescence, were identified in pemphigus patients. Post-diagnosis, only three neoplasm cases were identified, two of which were benign (Table 5).

**Table 4:** Association between autoimmune bullous disease (AIBD) and comorbidities in China, 2016–2023

Comorbidities	AIBD (*n* = 1072)		Pemphigus (*n* = 573)	Pemphigoid (*n* = 499)	*P*
Diabetes mellitus	166(15.49)		82(14.31)	84(16.83)	0.255
Type 2 diabetes	113(10.54)		48(8.38)	65(13.03)	0.013
Steroid diabetes	55(5.13)		36(6.28)	19(3.81)	0.067
Cardiovascular disease	367(34.24)		160(27.92)	207(41.48)	< 0.001
Hypertension	282(26.31)		122(21.29)	160(32.06)	< 0.001
Cardiac disease	126(11.75)		56(9.77)	70(14.03)	0.031
Coronary heart disease	77(7.18)		35(6.11)	42(8.42)	0.144
Myocardial ischemia	4(0.37)		3(0.52)	1(0.20)	0.387
Arrhythmia	69(6.44)		29(5.06)	40(8.02)	0.049
Valvular heart disease	6(0.56)		2(0.35)	4(0.80)	0.322
Cerebrovascular disease	96(8.96)		27(4.71)	69(13.83)	< 0.001
Cerebral hemorrhage	13(1.21)		4(0.70)	9(1.80)	0.099
Cerebral infarction	87(8.12)		23(4.01)	64(12.83)	< 0.001
Deep venous thrombosis	15(1.40)		6(1.05)	9(1.80)	0.293
Pulmonary disease	184(17.16)		90(15.71)	94(18.84)	0.175
Pulmonary bacterial/virus infection	60(5.60)		28(4.89)	32(6.41)	0.278
Pulmonary fungal infection	25(2.33)		14(2.44)	11(2.20)	0.796
Interstitial lung disease	60(5.60)		32(5.58)	28(5.61)	0.985
Pulmonary tuberculosis	37(3.45)		16(2.79)	21(4.21)	0.205
Pulmonary embolism	7(0.65)		4(0.70)	3(0.60)	0.844
Bronchitis	13(1.21)		5(0.87)	8(1.60)	0.276
Asthma	17(1.59)		3(0.52)	14(2.81)	0.003
Digestive system disease	113(10.54)		56(9.77)	57(11.42)	0.38
Digestive tract inflammation	76(7.09)		39(6.81)	37(7.41)	0.699
liver disease	44(4.10)		19(3.32)	25(5.01)	0.163
Renal disease	29(2.71)		10(1.75)	19(3.81)	0.038
Neurologic disease	53(4.94)		13(2.27)	40(8.02)	< 0.001
Cryptococcus meningitis	7(0.65)		3(0.52)	4(0.80)	0.573
Dementia	21(1.96)		3(0.52)	18(3.61)	< 0.001
Parkinson’s disease	15(1.40)		6(1.05)	9(1.80)	0.293
Poliomyelitis	2(0.19)		0(0.00)	2(0.40)	0.129
Encephalatrophy	17(1.59)		4(0.70)	13(2.61)	0.013
Psychiatric disease	14(1.31)		6(1.05)	8(1.60)	0.424
Mental disorder	9(0.84)		5(0.87)	4(0.80)	0.899
Schizophrenia	5(0.47)		1(0.17)	4(0.80)	0.133
Thyroid disease	33(3.08)		17(2.97)	16(3.21)	0.821
Psoriasis	15(1.40)		2(0.35)	13(2.61)	0.002
Ophthalogic Disease	129(12.03)		76(13.26)	53(10.62)	0.185
Osteonecrosis	25(2.33)		17(2.97)	8(1.60)	0.14

**Table 5:** Association between autoimmune bullous diseases (AIBD) and cancers in China, 2016–2023

Cancers	AIBD (*n* = 1072)		Pemphigus (*n* = 573)	Pemphigoid (*n* = 499)	*P*
Gastrointestinal cancer	23(2.15)		9(1.57)	14(2.81)	0.164
lung cancer	19(1.77)		4(0.70)	15(3.01)	0.004
Genitourinary cancer	11(1.03)		1(0.17)	10(2.00)	0.003
Gynecological cancer	9(0.84)		3(0.52)	6(1.20)	0.224
Thyroid cancer	5(0.47)		2(0.35)	3(0.60)	0.877
Thymic carcinoma	6(0.56)		5(0.87)	1(0.20)	0.289
Castleman’s disease	2(0.19)		2(0.35)	0(0.00)	0.541
Other	4(0.37)		2(0.35)	2(0.40)	1

### Mortality and Causes of Death

**Fig. 3:**
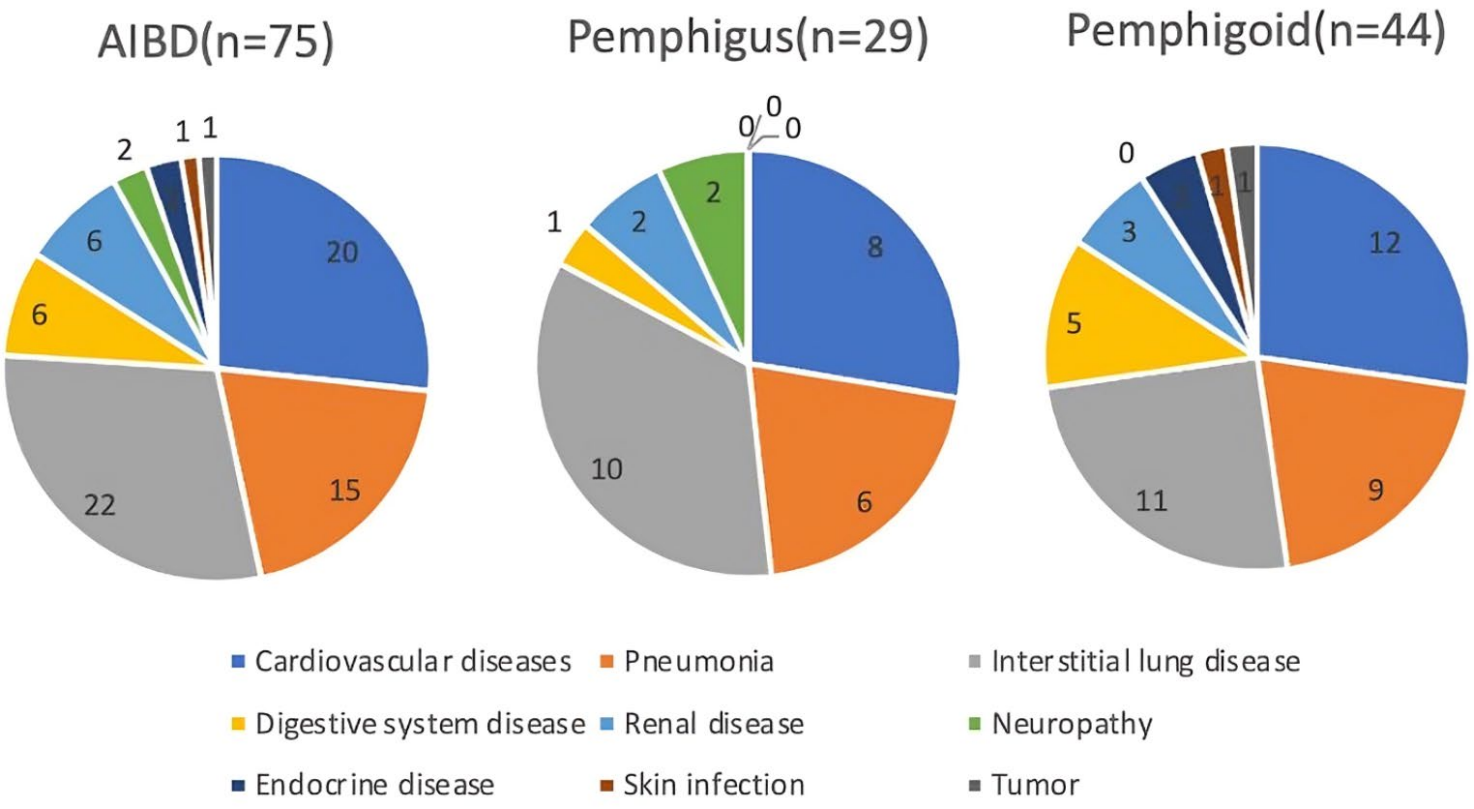
The cause of death for patients with autoimmune bullous disease patients in China, 2016–2023. The pie charts present the distribution of the cause of death among patients with different autoimmune bullous diseases: AIBD (Autoimmune Bullous Diseases), Pemphigus, and Pemphigoid. For AIBD (*n* = 75), cardiovascular diseases are the most common cause of death (20 cases), followed by digestive system disease (22 cases) and endocrine disease (15 cases). In Pemphigus (*n* = 29), cardiovascular diseases (10 cases) and skin infection (8 cases) are predominant, while for Pemphigoid (*n* = 44), the leading cause of death are cardiovascular diseases (12 cases) and interstitial lung disease (9 cases). Other notable conditions across the groups include pneumonia, renal disease, neuropathy, and tumors. The total number of patients within each disease category is indicated in parentheses

During the study, 59 patients (5.5% of the total cohort) passed away, with the majority of deaths (67.79%) occurring within the first year of autoimmune bullous disease diagnosis. The average survival duration post-diagnosis was significantly different between groups: 1.24 years (SD ± 0.84) for pemphigus and 0.76 years (SD ± 0.68) for pemphigoid (*p* < 0.05). Pemphigoid had a higher mortality rate (7.41%) compared to pemphigus (3.84%, *p* < 0.05). The mortality rates at 1, 2, and 3 years were 3.7%, 4.9%, and 5.3%, respectively. A significant difference was also observed in the mean age at death (pemphigus: 62.75 years, SD ± 14.35; pemphigoid: 76.41 years, SD ± 9.61; *p* < 0.001) (Table 2). The standardized mortality ratio (SMR) varied significantly by age group, with the highest SMR for pemphigus (25) in the 70–79 age group and for pemphigoid (100) in the over-80 age group, attributed to a higher-than-expected deceased patient count in this demographic (Table 6). The leading causes of death were interstitial lung disease (29.33%), cardiovascular diseases (22.67%), and pneumonia (20%) (Fig. 3).

**Table 6:** The mortality rate (MR) and standardized mortality rate (SMR) in autoimmune bullous diseases (AIBD) patients by different age groups in China, 2016–2023

Age (Years)	AIBD patients				The Sixth National Census in China (*n* = 1,332,810,869)			Standardized AIBD patients	
	Dead patients	Total patients	Mortality (%)		Population	proportion (%)		Expected total patients	Standardized mortality (%)
AIBD patients (*n* = 1072)									
< 40	2	140	1.43		764,802,267	57.38		615	0.33
40–49	4	143	2.80		230,348,517	17.28		185	2.16
50–59	5	195	2.56		160,065,645	12.09		130	3.85
60–69	15	259	5.79		99,780,564	7.49		80	18.75
70–79	17	196	8.67		56,824,530	4.26		46	36.96
≥ 80	16	139	11.51		20,989,346	1.57		17	94.12
Pemphigus (*n* = 573)									
< 40	2	109	1.83		764,802,267	57.38		329	0.61
40–49	4	115	3.48		230,348,517	17.28		99	4.04
50–59	3	125	2.40		160,065,645	12.09		69	4.35
60–69	5	136	3.68		99,780,564	7.49		43	11.63
70–79	6	60	10.00		56,824,530	4.26		24	25.00
≥ 80	2	28	7.14		20,989,346	1.57		9	22.22
Pemphigoid (*n* = 499)									
< 40	0	31	0.00		764,802,267	57.38		286	0.00
40–49	0	28	0.00		230,348,517	17.28		86	0.00
50–59	2	70	2.86		160,065,645	12.09		60	3.33
60–69	10	123	8.13		99,780,564	7.49		37	27.03
70–79	11	136	8.09		56,824,530	4.26		21	52.38
≥ 80	14	111	12.61		20,989,346	1.57		8	100.00 ‡

‡: the mortality rate in the standardized patients population was set as 100% due to the number of dead patients exceeded the number of expected total patients after the standardization in this group)

### Risk Factors for Mortality among Autoimmune Bullous Disease Patients

Kaplan-Meier univariate and Cox regression univariate analyses indicated that pemphigoid is associated with a worse prognosis compared to pemphigus (*p* = 0.006 and *p* = 0.007, respectively). Older age and male gender were linked to poorer outcomes (*p* = 0.000 and *p* < 0.001 for age; *p* = 0.029 and *p* = 0.031 for gender, respectively). Multiple comorbidities were a significant influencing factor (*p* < 0.001) (Table 7). Specific comorbidities, including diabetes, hypertension, coronary heart disease, arrhythmia, heart valvular disease, cerebral hemorrhage, cerebral infarction, deep venous thrombosis, pulmonary bacterial/virus infection, pulmonary fungal infection, interstitial lung disease, renal disease, poliomyelitis, and encephalatrophy, were prognostic indicators of poor survival. Cox multivariable regression revealed that pulmonary bacterial/virus infection (HR 13.12, 95% CI 7.57–22.76, *p* < 0.001), interstitial lung disease (HR 7.28, 95% CI 4.12–12.85, *p* < 0.001), arrhythmia (HR 5.17, 95% CI 2.74–9.75, *p* < 0.001), pulmonary fungal infection (HR 3.8, 95% CI 1.55–9.31, *p* < 0.01), coronary heart disease (HR 1.96, 95% CI 1.00-3.84, *p* < 0.05), and pemphigoid (HR 1.85, 95% CI 1.09–3.15, *p* < 0.05) significantly affected prognosis (Table 8).

**Table 7:** The potential influencing factors for the mortality of autoimmune bullous diseases (AIBD) patients in China by univariate analysis, 2016–2023

Variables	Kaplan-Meier (Log-rank test)				Cox Regression		
	Means for ST† (year)	95% CI of Means	*p*		HR	95% CI	*p*
AIBD types			0.006				0.007
Pemphigus	22.29	21.93–22.65			1.00	-	
Pemphigoid	17.73	17.29–18.18			**2.07**	**1.22–3.51**	
Gender			0.029				0.031
Male	19.70	19.27–20.14			**1.82**	**1.06–3.15**	
Female	22.28	21.89–22.67			1.00	-	
Age (years)			0.000				0.001
<40	17.92	17.57–18.26			1.00	-	
40–49	14.03	13.66–14.40			1.97	0.36–10.73	0.435
50–59	22.58	22.07–23.09			1.83	0.36–9.45	0.469
60–69	19.92	19.31–20.53			4.23	0.97–18.49	0.055
70–79	12.12	11.61–12.63			6.61	1.53–28.62	0.012
≥80	13.80	12.98–14.62			9.19	2.11–39.98	0.003
Multiple comorbidity			0.000				0.000
Yes	14.77	13.74–15.79			**8.07**	**4.78–13.62**	
No	22.57	22.32–22.81			1.00	-	

†:ST for Survival Time

CI: Confidence Interval

**Table 8:** The risk factors for mortality of autoimmune bullous diseases (AIBD) patients in China by Cox multivariable analysis, 2016–2023

Variables	HR		95% CI	*P*
Pemphigoid	1.85		1.09–3.15	0.024
Coronary heart disease	1.96		1.00-3.84	0.049
Pulmonary fungal infection	3.80		1.55–9.31	0.004
Arrhythmia	5.17		2.74–9.75	0.000
Interstitial lung disease	7.28		4.12–12.85	0.000
Pulmonary bacterial/virus infection	13.12		7.57–22.76	0.000

## Discussion

Our extensive analysis of 1072 patients with autoimmune bullous disease, encompassing both pemphigus vulgaris and bullous pemphigoid, offers a comprehensive view of these conditions in a Chinese clinical setting. A significant observation is the gender and age correlation in autoimmune bullous disease incidence, with males and older individuals more frequently affected [22–25]. This demographic pattern is crucial for understanding the epidemiology of autoimmune bullous disease and tailoring patient-specific management strategies.

The mortality rate of 5.5% in autoimmune bullous disease, primarily due to interstitial lung disease, cardiovascular diseases, and pneumonia, is a stark reminder of the severity of these conditions. This mortality rate, along with identified risk factors such as pulmonary infections and arrhythmias, underscores the importance of proactive management of these comorbidities [9, 26, 27].

We noted significant socioeconomic implications of autoimmune bullous disease. The lower average annual income of patients, in comparison to Shanghai’s average, and a higher illiteracy rate in our patient cohort, underscore the economic and educational challenges faced by autoimmune bullous disease patients [28]. Individuals with limited financial means may struggle to access healthcare and adhere to treatment, leading to delayed diagnosis and suboptimal management. Similarly, higher illiteracy rates may hinder understanding of the disease and impede effective self-management strategies, exacerbating the disease burden. Addressing these socioeconomic factors through targeted support programs, such as financial assistance and health literacy initiatives, is crucial for improving disease management and patient outcomes in autoimmune bullous disease. By recognizing these broader implications, policymakers and healthcare providers can develop more effective strategies to alleviate the burden of the disease.

The occupational risks associated with autoimmune bullous disease, particularly in farmers and metal workers, emphasize the potential role of environmental factors in disease etiology. In addition, we found that the mortality rate of patients was higher in spring and summer. Compared with other articles, some found that pemphigus was more common in spring and summer [29, 30], while others said that it was not related to the season [31]. The exposure to UV light, extreme temperatures, and possibly other occupational hazards could be contributing to the higher incidence and mortality rates in these groups [32]. This finding is critical for developing workplace health policies and preventive strategies to protect at-risk populations. For example, autoimmune bullous disease patients should avoid prolonged exposure to sunlight and heat, and refrain from occupations involving exposure to light or heat. Conversely, individuals engaged in high-risk occupations should take proactive measures to protect themselves from light and heat.

In terms of comorbidities, our study adds to the growing body of evidence linking autoimmune bullous disease with neurological disorders. The significant incidence of neuropathy in pemphigoid patients and the development of conditions like Alzheimer’s disease and Parkinson’s disease prior to autoimmune bullous disease onset suggest a complex interplay between neurodegenerative processes and autoimmune bullous disease [33–35]. This interconnection warrants further investigation into the shared pathogenic mechanisms, which could lead to novel therapeutic approaches.

The relationship between autoimmune bullous disease and cancer observed in our study aligns with some global studies while contrasting with others, indicating a need for further research to unravel this complex association [36, 37]. Understanding the link between cancer and autoimmune bullous disease could have significant implications for patient screening, management, and treatment strategies. The shift in comorbid disease prevalence post-autoimmune bullous disease diagnosis, particularly towards pulmonary diseases, underscores the importance of comprehensive patient management strategies. These should include monitoring for complications arising from long-term immunosuppressant use and addressing the increased risk of infections, especially respiratory infections [9].

Our study challenges the conventional view of bullous diseases as organ-specific disorders. The findings suggest that autoimmune bullous disease might be systemic, affecting multiple organs, much like systemic sclerosis, systemic lupus erythematosus, and polymyositis/dermatomyositis [38, 39]. The notable incidence of interstitial lung disease in autoimmune bullous disease patients, with no significant difference between pemphigus and pemphigoid, is a pioneering observation in our research, emphasizing the need for in-depth investigation into the link between interstitial lung disease and autoimmune bullous disease.

The mechanism between interstitial lung disease and autoimmune bullous disease is unknown. In a case report of pemphigoid with interstitial lung disease, linear deposits of IgG and C3 were found in the alveolar basement membrane by direct immunofluorescence on a lung biopsy [40]. It has been reported that IgG deposition in respiratory epithelial tissue can be seen in autoimmune bullous disease patients with lung injury [41]. These cases suggest that lung may be the target organ of autoimmune bullous disease, and the autoimmune reaction with autoantibodies may be the cause of interstitial lung disease. However, its pathological mechanism still needs to be further studied and verified.

In conclusion, our extensive single-center cross-sectional study provides valuable insights into autoimmune bullous disease in the Chinese context. It underscores the complexity of autoimmune bullous disease as a disease that interweaves clinical, demographic, socioeconomic, and environmental factors. Our findings advocate for a holistic approach to management and treatment, considering not only the medical but also the socioeconomic aspects of the disease. The study opens numerous avenues for future research, especially in understanding the systemic nature of autoimmune bullous disease and its association with other diseases, and in developing more effective and safer treatment modalities.

### Limitation

One limitation of our study is its single-center design, which may not fully represent the diverse population of autoimmune bullous diseases patients in China. Furthermore, the use of face-to-face interviews for data collection might have introduced response bias. To overcome these limitations and obtain a more comprehensive understanding of autoimmune bullous disease across the broader Chinese demographic, future studies should consider employing multicenter cohort or case-control approaches.

## Conclusion

This study represents a significant contribution to the understanding of autoimmune bullous disease in a Chinese clinical setting, marking it as one of the first extensive single-center cross-sectional studies in this domain. Our comprehensive analysis sheds light on the complex interplay of comorbidities, mortality, risk factors, and socioeconomic factors associated with autoimmune bullous disease in China.

A notable finding is the high prevalence of autoimmune bullous disease among workers in industries requiring occupational protection. This underscores the urgent need for enhanced safety measures and increased awareness in these sectors to mitigate the risk of developing autoimmune bullous disease. Furthermore, our study reveals a considerable proportion of autoimmune bullous disease patients with limited literacy and from low-income backgrounds, highlighting the significant economic impact on patients, their families, and the broader society. These findings emphasize the importance of developing targeted support systems and healthcare policies that address both the medical and socioeconomic challenges faced by autoimmune bullous disease patients.

Additionally, our research makes a pivotal contribution by exploring the association between autoimmune bullous disease and interstitial lung disease. This aspect of the study not only provides new insights into the clinical profile of autoimmune bullous disease but also opens avenues for future research to investigate the underlying pathological mechanisms linking autoimmune bullous disease and interstitial lung disease. Such research could potentially lead to more effective treatment strategies and improved patient outcomes.

The comorbidity screening and socioeconomic burden highlighted in our study indicate a need for healthcare support. During follow-up periods, monitoring cardiopulmonary health, including cardiopulmonary auscultation at each visit and regular lung CT screenings every 3–6 months, is essential. Future research efforts should focus on exploring the pathogenic mechanisms between interstitial lung disease and autoimmune bullous disease. Further validation could be achieved through establishing mouse models and conducting immunopathological experiments.

In conclusion, our study offers a comprehensive overview of autoimmune bullous disease in the Chinese population, presenting valuable data that can inform clinical practice and guide future research. It lays a foundation for a deeper understanding of the disease and its broader implications, ultimately contributing to better management and care for patients with autoimmune bullous disease.

## Electronic supplementary material

Below is the link to the electronic supplementary material.

Supplementary Material 1

## Data Availability

No datasets were generated or analysed during the current study.
