# Supplementary material for: Epidemiological Insights into Autoimmune Bullous Diseases in China: A Comprehensive Analysis
Source: J Epidemiol Glob Health. 2024 Jul 22;14(3):513–23. doi: 10.1007/s44197-024-00277-7 (PMC11442409; doi:10.1007/s44197-024-00277-7)
Supplement: Supplementary file 1 — Supplementary Material 1 [file 44197_2024_277_MOESM1_ESM.docx]

**Table E1. Comorbidities prior to the onset of AIBD in China, 2016-2023.**

| Comorbidities | AIBD  (n=1072) |  | Pemphigus  (n=573) | Pemphigoid  (n=499) | *P* |
| --- | --- | --- | --- | --- | --- |
| Diabetes mellitus | 110(10.26) |  | 47(8.20) | 63(12.63) | 0.017 |
| Type 2 diabetes | 110(10.26) |  | 47(8.20) | 63(12.63) | 0.017 |
| Cardiovascular disease | 329(30.69) |  | 138(24.08) | 191(38.28) | <0.001 |
| Hypertension | 255(23.79) |  | 107(18.67) | 148(29.66) | <0.001 |
| Cardiac disease | 105(9.79) |  | 47(8.20) | 58(11.62) | 0.06 |
| Coronary heart disease | 68(6.34) |  | 31(5.41) | 37(7.41) | 0.179 |
| Myocardial ischemia | 4(0.37) |  | 3(0.52) | 1(0.20) | 0.387 |
| Arrhythmia | 50(4.66) |  | 21(3.66) | 29(5.81) | 0.096 |
| Valvular heart disease | 5(0.47) |  | 2(0.35) | 3(0.60) | 0.546 |
| Cerebrovascular disease | 85(7.93) |  | 21(3.66) | 64(12.83) | <0.001 |
| Cerebral hemorrhage | 12(1.12) |  | 4(0.70) | 8(1.60) | 0.16 |
| Cerebral infarction | 77(7.18) |  | 17(2.97) | 60(12.02) | <0.001 |
| Deep venous thrombosis | 3(0.28) |  | 2(0.35) | 1(0.20) | 0.646 |
| Pulmonary disease | 57(5.32) |  | 24(4.19) | 33(6.61) | 0.078 |
| Pulmonary bacterial/virus infection | 1(0.09) |  | 1(0.17) | 0(0.00) | 0.35 |
| Pulmonary fungal infection | 1(0.09) |  | 0(0.00) | 1(0.20) | 0.284 |
| Interstitial lung disease | 2(0.19) |  | 1(0.17) | 1(0.20) | 0.922 |
| Pulmonary tuberculosis | 28(2.61) |  | 13(2.27) | 15(3.01) | 0.45 |
| Pulmonary embolism | 2(0.19) |  | 1(0.17) | 1(0.20) | 0.922 |
| Bronchitis | 13(1.21) |  | 5(0.87) | 8(1.60) | 0.276 |
| Asthma | 16(1.49) |  | 3(0.52) | 13(2.61) | 0.005 |
| Digestive system disease | 95(8.86) |  | 46(8.03) | 49(9.82) | 0.303 |
| Digestive tract inflammation | 59(5.50) |  | 28(4.89) | 31(6.21) | 0.342 |
| liver disease | 41(3.82) |  | 19(3.32) | 22(4.41) | 0.352 |
| Renal disease | 24(2.24) |  | 9(1.57) | 15(3.01) | 0.113 |
| Neurologic disease | 40(3.73) |  | 7(1.22) | 33(6.61) | <0.001 |
| Dementia | 20(1.87) |  | 2(0.35) | 18(3.61) | <0.001 |
| Parkinson's disease | 12(1.12) |  | 3(0.52) | 9(1.80) | 0.047 |
| Poliomyelitis | 2(0.19) |  | 0(0.00) | 2(0.40) | 0.129 |
| Encephalatrophy | 13(1.21) |  | 3(0.52) | 10(2.00) | 0.027 |
| Psychiatric disease | 10(0.93) |  | 4(0.70) | 6(1.20) | 0.392 |
| Mental disorder | 5(0.47) |  | 3(0.52) | 2(0.40) | 0.769 |
| Schizophrenia | 5(0.47) |  | 1(0.17) | 4(0.80) | 0.133 |
| Thyroid disease | 27(2.52) |  | 13(2.27) | 14(2.81) | 0.576 |
| Psoriasis | 15(1.40) |  | 2(0.35) | 13(2.61) | 0.002 |
| Ophthalogic Disease | 40(3.73) |  | 14(2.44) | 26(5.21) | 0.017 |
| Osteonecrosis | 2(0.19) |  | 2(0.35) | 0(0.00) | 0.187 |

**Table E2. Comorbidities following the onset of AIBD in China, 2016-2023.**

| Comorbidities | AIBD  (n=1072) |  | Pemphigus  (n=573) | Pemphigoid  (n=499) | *P* |
| --- | --- | --- | --- | --- | --- |
| Diabetes mellitus | 59(5.50) |  | 38(6.63) | 21(4.21) | 0.083 |
| Type 2 diabetes | 4(0.37) |  | 2(0.35) | 2(0.40) | 0.89 |
| Steroid diabetes | 55(5.13) |  | 36(6.28) | 19(3.81) | 0.067 |
| Cardiovascular disease | 60(5.60) |  | 31(5.41) | 29(5.81) | 0.775 |
| Hypertension | 30(2.80) |  | 15(2.62) | 15(3.01) | 0.701 |
| Cardiac disease | 26(2.43) |  | 11(1.92) | 15(3.01) | 0.249 |
| Coronary heart disease | 12(1.12) |  | 4(0.70) | 8(1.60) | 0.16 |
| Arrhythmia | 21(1.96) |  | 9(1.57) | 12(2.40) | 0.326 |
| Valvular heart disease | 1(0.09) |  | 0(0.00) | 1(0.20) | 0.284 |
| Cerebrovascular disease | 11(1.03) |  | 6(1.05) | 5(1.00) | 0.942 |
| Cerebral hemorrhage | 1(0.09) |  | 0(0.00) | 1(0.20) | 0.284 |
| Cerebral infarction | 10(0.93) |  | 6(1.05) | 4(0.80) | 0.677 |
| Deep venous thrombosis | 12(1.12) |  | 4(0.70) | 8(1.60) | 0.16 |
| Pulmonary disease | 139(12.97) |  | 70(12.22) | 69(13.83) | 0.433 |
| Pulmonary bacterial/virus infection | 59(5.50) |  | 27(4.71) | 32(6.41) | 0.223 |
| Pulmonary fungal infection | 24(2.24) |  | 14(2.44) | 10(2.00) | 0.628 |
| Interstitial lung disease | 58(5.41) |  | 31(5.41) | 27(5.41) | 1 |
| Pulmonary tuberculosis | 10(0.93) |  | 4(0.70) | 6(1.20) | 0.392 |
| Pulmonary embolism | 5(0.47) |  | 3(0.52) | 2(0.40) | 0.769 |
| Bronchitis | 1(0.09) |  | 1(0.17) | 0(0.00) | 0.35 |
| Asthma | 1(0.09) |  | 0(0.00) | 1(0.20) | 0.284 |
| Digestive system disease | 22(2.05) |  | 12(2.09) | 10(2.00) | 0.917 |
| Digestive tract inflammation | 17(1.59) |  | 11(1.92) | 6(1.20) | 0.348 |
| liver disease | 5(0.47) |  | 1(0.17) | 4(0.80) | 0.133 |
| Renal disease | 5(0.47) |  | 1(0.17) | 4(0.80) | 0.133 |
| Neurologic disease | 13(1.21) |  | 6(1.05) | 7(1.40) | 0.596 |
| Cryptococcus meningitis | 7(0.65) |  | 3(0.52) | 4(0.80) | 0.573 |
| Dementia | 1(0.09) |  | 1(0.17) | 0(0.00) | 0.35 |
| Parkinson's disease | 3(0.28) |  | 3(0.52) | 0(0.00) | 0.106 |
| Encephalatrophy | 4(0.37) |  | 1(0.17) | 3(0.60) | 0.253 |
| Psychiatric disease | 4(0.37) |  | 2(0.35) | 2(0.40) | 0.89 |
| Mental disorder | 4(0.37) |  | 2(0.35) | 2(0.40) | 0.89 |
| Thyroid disease | 6(0.56) |  | 4(0.70) | 2(0.40) | 0.515 |
| Psoriasis | 1(0.09) |  | 0(0.00) | 1(0.20) | 0.284 |
| Ophthalogic Disease | 90(8.40) |  | 63(10.99) | 27(5.41) | 0.001 |
| Osteonecrosis | 23(2.15) |  | 15(2.62) | 8(1.60) | 0.253 |
